# Ex Vivo Expanded and Activated Natural Killer Cells Prolong the Overall Survival of Mice with Glioblastoma-like Cell-Derived Tumors

**DOI:** 10.3390/ijms22189975

**Published:** 2021-09-15

**Authors:** Yoichi Shida, Tsutomu Nakazawa, Ryosuke Matsuda, Takayuki Morimoto, Fumihiko Nishimura, Mitsutoshi Nakamura, Ryosuke Maeoka, Shuichi Yamada, Ichiro Nakagawa, Young-Soo Park, Motoaki Yasukawa, Takashi Tojo, Takahiro Tsujimura, Hiroyuki Nakase

**Affiliations:** 1 Department of Neurosurgery, Nara Medical University, Kashihara 634-8521, Nara, Japan; yoichi_0723@yahoo.co.jp (Y.S.); nakazawa@naramed-u.ac.jp (T.N.); t.morimoto@naramed-u.ac.jp (T.M.); fnishi@naramed-u.ac.jp (F.N.); mnaka@grandsoul.co.jp (M.N.); r.maeoka@naramed-u.ac.jp (R.M.); syamada@naramed-u.ac.jp (S.Y.); nakagawa@naramed-u.ac.jp (I.N.); park-y-s@naramed-u.ac.jp (Y.-S.P.); nakasehi@naramed-u.ac.jp (H.N.); 2 Grandsoul Research Institute for Immunology, Inc., Uda 633-2221, Nara, Japan; 3 Clinic Grandsoul Nara, Uda 633-2221, Nara, Japan; takahiro@grandsoul.co.jp; 4 Department of Thoracic Surgery, Nara Medical University, Kashihara 634-8521, Nara, Japan; myasu1007@hotmail.com (M.Y.); tcaytojo@gmail.com (T.T.); 5 Department of Surgery, Osaka Kaisei Hospital, Yodogawa, Osaka 532-0003, Japan; 6 Department of Thoracic Surgery, Saiseikai Chuwa Hospital, Sakurai 633-0054, Nara, Japan

**Keywords:** PD-1, PD-L1, NK cell, glioblastoma

## Abstract

Glioblastoma (GBM) is the leading malignant intracranial tumor and is associated with a poor prognosis. Highly purified, activated natural killer (NK) cells, designated as genuine induced NK cells (GiNKs), represent a promising immunotherapy for GBM. We evaluated the anti-tumor effect of GiNKs in association with the programmed death 1(PD-1)/PD-ligand 1 (PD-L1) immune checkpoint pathway. We determined the level of PD-1 expression, a receptor known to down-regulate the immune response against malignancy, on GiNKs. PD-L1 expression on glioma cell lines (GBM-like cell line U87MG, and GBM cell line T98G) was also determined. To evaluate the anti-tumor activity of GiNKs in vivo, we used a xenograft model of subcutaneously implanted U87MG cells in immunocompromised NOG mice. The GiNKs expressed very low levels of PD-1. Although PD-L1 was expressed on U87MG and T98G cells, the expression levels were highly variable. Our xenograft model revealed that the retro-orbital administration of GiNKs and interleukin-2 (IL-2) prolonged the survival of NOG mice bearing subcutaneous U87MG-derived tumors. PD-1 blocking antibodies did not have an additive effect with GiNKs for prolonging survival. GiNKs may represent a promising cell-based immunotherapy for patients with GBM and are minimally affected by the PD-1/PD-L1 immune evasion axis in GBM.

## 1. Introduction

Glioblastoma (GBM) is the most common primary malignant tumor of the brain. Although standard treatment is comprised of maximal surgical resection followed by adjuvant radiotherapy and chemotherapy with temozolomide (TMZ), the median survival time remains less than 2 years [1,2]. Bevacizumab is a humanized antibody that inhibits vascular endothelial growth factor, and may improve patient status and reduce the use of corticosteroids; however, a previous clinical trial did not report a significantly longer overall survival time in patients with newly diagnosed GBM [3]. Therefore, there is an urgent need for the identification of novel and effective treatment strategies for GBM.

Natural killer (NK) cells comprise less than 15% of all human peripheral blood lymphocytes and are defined phenotypically based on their expression of CD56 (neural cell adhesion molecule) and lack of CD3 expression (part of the T cell receptor complex) [4]. Moreover, NK cells are crucial components of the innate immune system and can recognize abnormal cells, including transformed cells or virus-infected cells, without prior sensitization [5,6]. NK cells also exhibit potent cytotoxic activity against tumor cells by promoting apoptosis [7] and can remove abnormal cells involved in the innate immune system [8,9]. NK cells recognize tumor cells by forming a synapse with their receptors and induce apoptosis through the release of perforin and granzymes, cytotoxic molecules directed against the target cells of the tumor [10]. Perforin functions by inducing the formation of pores on the tumor, through which granzymes can be delivered into the tumor cells [11]. Subsequently, granzyme-activated caspase induces tumor cell death via apoptosis [12]. The cytotoxic function of NK cells is determined by the balance between activating and inhibitory receptor signals [13,14]. Several activating receptors expressed by NK cells recognize the associated ligands expressed on GBM cells [15,16], and the ligation of the activating receptors triggers cytotoxicity in NK cells [17]. Natural cytotoxicity triggering receptor 1 (NCR1) is a representative receptor of NK cells and activates NK cells by recognizing the specific ligand. The ligands of NK inhibitory receptors are also associated with NK cell-mediated cytotoxicity against tumor cells [18,19,20]. In addition, NK cells produce immunoregulatory cytokines (i.e., IL-2 and IFN-γ) and modulate the immune response towards antitumor or antivirus immunity [21]. NK cells play a role in immune surveillance and inhibit cancer tumor occurrence, proliferation, and metastasis [22,23]. Thus, there is considerable interest in harnessing these properties of NK cells for the next wave of cancer immunotherapy [24].

Multiple clinical studies have validated the use of NK cells as a promising therapeutic option for the treatment of malignant tumors [25,26]. The efficacy of adoptively transferred autologous lymphokine-activated killer cells (LAKs) has also been comprehensively explored since the earliest studies in the late 1980s [27]. Treatment with local autologous LAKs was reported to be safe and have shown a survival benefit [28]. Despite these findings, the clinical applications of NK cell-based immunotherapy, especially for GBM, have scarcely been reported due to challenges in the large-scale expansion of highly purified and stably activated NK cells [29]. In addition, immunosuppressive T cell components, such as regulatory T cells (Tregs), in the LAK population can also suppress the cytolytic activity of NK cells [30].

Recent studies have demonstrated that immune checkpoint blockers (ICBs) have been attracting increased attention as a promising treatment strategy for a variety of malignancies. ICBs upregulate the function of immune cells against cancer cells, including malignant melanoma and non-small cell lung cancer [31]. GBM has been found to exhibit significantly higher levels of PD-L1 expression compared to that of lower-grade glioma [32]. Miyazaki et al. demonstrated that high PD-1 expression in recurrent GBM was associated with a shorter survival time following secondary resection [33]. In a recent Phase 3 randomized clinical trial for recurrent GBM treated with bevacizumab or nivolumab (an anti PD-1 antibody), nivolumab failed to demonstrate superiority. However, patients with methylated-O6-methylguanine DNA-methyltransferase (MGMT) promoter glioblastoma and no baseline corticosteroid use may potentially benefit from treatment with ICBs [34].

Our previous research focused on the function of NK cells against GBM cells, and widely discussed the potential use of NK cells as an immunotherapy for GBM. We established highly purified, activated natural killer (NK) cells, designated as genuine induced NK cells (GiNKs), using a novel ex vivo expanding culture method. Our results indicated that GiNKs represent a promising alternative for cell-based immunotherapy for GBM [35,36]. The present study aimed to further examine the anti-tumor effects of the GiNKs against GBM cell lines in vitro, and intravenously transferring GiNKs to a subcutaneous GBM-like xenograft model in vivo. Second, we evaluated the involvement of the PD-1/PD-L1 pathway using PD-1 blockers following immunotherapy with GiNKs against GBM cell lines, and in a GBM-like xenograft model in vivo.

## 2. Results

### 2.1. PD-1/PD-L1 Expression and NCR1 Based on the TCGA Data Set

**Figure 1:**
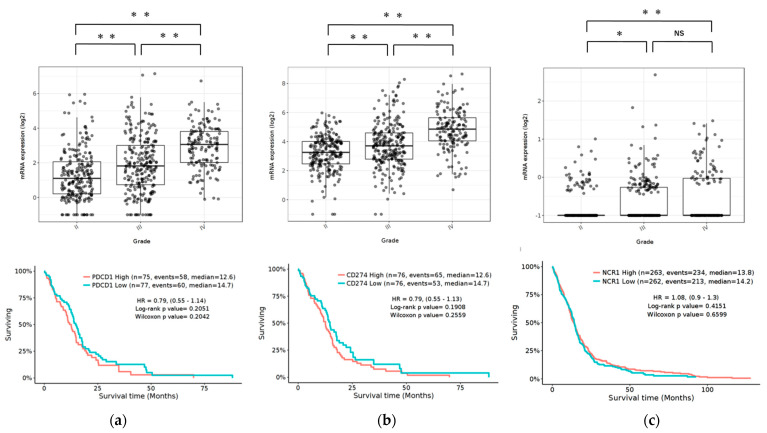
Expression of PD-1, PD-L1, and NCR1 in gliomas in the TCGA data set. **Top**: mRNA expression of PD-1 (**a**), PD-L1 (**b**) and NCR1 (**c**) according to the WHO grading system. **Bottom**: Kaplan-Meier curves based on mRNA expression. *p* Values were determined using Turkey’s Honest significant difference test. ** *p* < 0.001, * *p* < 0.01, NS: not significant.

To determine the expression pattern of the PD-1/PD-L1 pathway and NCR1 in gliomas, we examined the RNA-sequencing data of gliomas from the GlioVis data portal to visualize and analyze brain tumor expression datasets [37]. Our results revealed that PD-1 and PD-L1 were expressed in GBM in The Cancer Genome Atlas (TCGA) database. Compared to WHO grade II and grade III glioma, GBM (grade IV) was associated with the highest level of PD-1/PD-L1 expression (*p* < 0.05). Furthermore, NCR1, a representative receptor of NK cells, was also expressed in GBM, albeit at quite a low level. Compared to grade II, GBM had significant expression (*p* < 0.05). The expression of PD-1/PD-L1 and NCR1 was not associated with any differences in the overall survival (OS) (Figure 1).

### 2.2. Expression of PD-L1 in GBM Cell Lines

**Figure 2:**
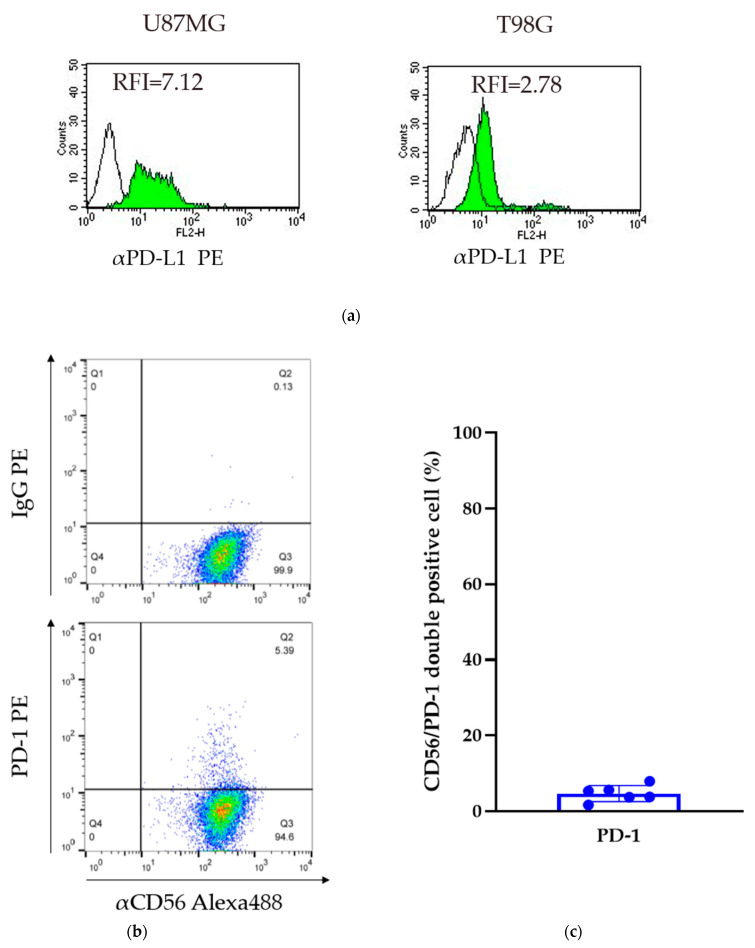
Expression of PD-L1 in GBM cell lines and PD-1 on GiNKs. (**a**) The relative Figure 1 was calculated based on the fluorescent intensity of PE-conjugated PD-L1 antibodies divided by the fluorescent intensity of PE-conjugated IgG. Left: U87MG cells and right: T98G cells. (**b**) The expression of PD-1 on the surface of GiNKs harvested from three healthy volunteers was detected. (**c**) The frequency of PD-1+/CD56+ NK cells of at least two independent experiments (*n* = 6).

Two glioma cell lines were obtained (U87MG and T98G cells). The relative fluorescence intensity (RFI) of PD-L1 was assessed by comparing the levels of expression to that of a control IgG. The RFI of the U87MG and T98G cells was 7.12 and 2.78, respectively. In both cell lines, PD-L1 was expressed on the cell surface; however, the level of expression was variable (Figure 2a).

### 2.3. Expression of PD-1 on GiNKs

Flow cytometry analysis detected PD-1 expression on the surface of GiNKs harvested from healthy volunteers. The frequency of PD-1+/CD56+ NK cells ranged between 1.7% and 8.0% in three healthy individuals (*n* = 6). The percentage of PD-1-positive cells was highly variable among healthy volunteers, but was less than 10% in each of the samples (Figure 2b,c). A very low frequency of PD-1 expressing NK cells was included in the GiNKs. We demonstrated that γδT cells had a positive expression of PD-1 in Supplementary Figure S1.

### 2.4. Apoptosis-Inducing Effects of GiNKs on GBM Cell Lines In Vitro

**Figure 3:**
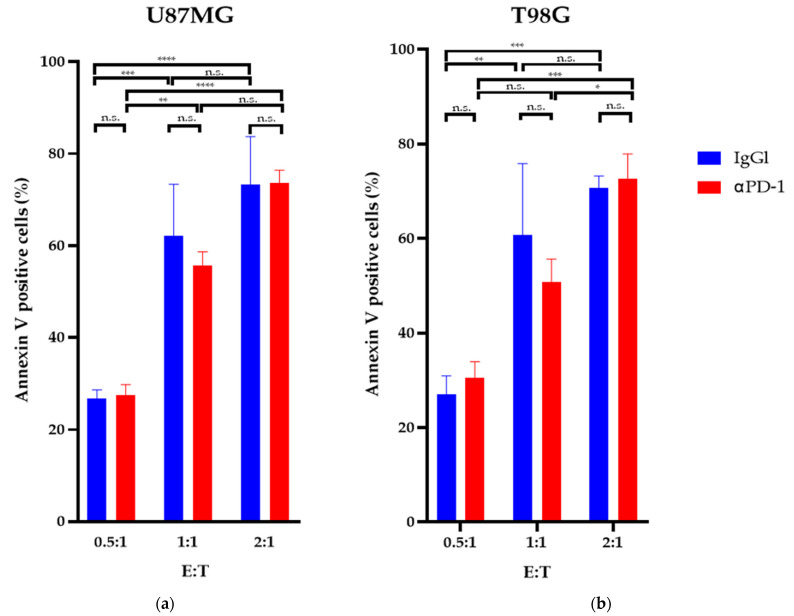
Apoptosis-inducing effects of GiNK on GBM cell lines in vitro. U87MG (**a**) and T98G (**b**) cell lines were exposed to GiNK at a GiNK:GBM cell ratio of 0.5:1, 1:1, and 2:1 with and without a PD-1 blocker (nivolumab). Nonadherent and detached adherent cells were stained with Annexin V–FITC, and the stained cells were analyzed using flow cytometry. Annexin V-positive cells are presented as a percentage of apoptotic cells from the total number of counted cells. The graphs show the percentages of Annexin V-positive apoptotic U87MG and T98G cells. Data are presented as the mean ± SE of at least two independent experiments (*n* = 3). *p* Values were determined using a two-way ANOVA followed by a Sidak’s test. **** *p* < 0.0001; *** *p* < 0.001; ** *p* < 0.01; * *p* < 0.05; n.s.: not significant.

The apoptosis detection assays revealed that after 24 h of exposure, a GiNK (effector) to GBM cell (target) ratio (E:T) of 2:1 significantly induced apoptosis in both GBM cell lines compared with an E:T ratio of 0.5:1 (*p* < 0.05). The GiNKs significantly induced apoptosis in U87MG and T98G cells (*p* < 0.05); however, treatment with the PD-1 blocker did not enhance or attenuate the apoptosis-inducing effect in both cell lines (Figure 3).

### 2.5. GiNKs Produce Cytokines upon Recognizing GBM Cells

**Figure 4:**
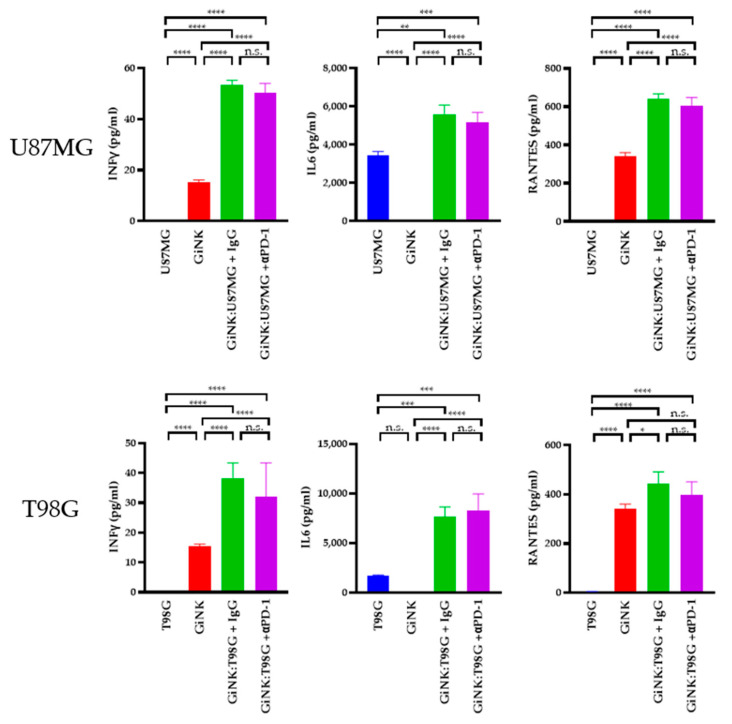
The cytokine production in GiNKs cultured with GBM cells. GBM cells (U87MG or T98G cell lines) were co-cultured alone and with GiNKs at the indicated ratios for 24 h. The concentrations of interferon-γ (IFNγ), interleukin-6 (IL-6), and regulated on activation, normal T-cells expressed and secreted (RANTES) in the supernatant were determined. Values are presented as the means ± SD at least two independent experiments (*n* = 3) in the upper table. Significant differences in lower figures were determined using a one-way analysis of variance (ANOVA), followed by a Tukey’s test. The figure represents table data. **** *p* < 0.0001; *** *p* < 0.001; ** *p* < 0.01; * *p* < 0.05; n.s.: not significant.

Next, we sought to determine the cytokine levels produced by GiNK (effector) cells following exposure to U87MG (target) and T98G (target) cells. The levels of interferon (IFN)γ in the supernatants of U87MG cells co-cultured with GiNKs were 53.4 ± 1.2 pg/mL and 50.3 ± 2.6 pg/mL (treated with the PD-1 blocker), respectively, using the same E:T ratios. Treatment with the PD-1 blocker did not induce the production of the IFNγ on U87MG. The IFNγ levels in the supernatants from T98G cells co-cultured with GiNKs were 38.2 ± 3.7 pg/mL and 32.1 ± 8.0 pg/mL (treated with the PD-1 blocker), respectively, using the same E:T ratios. In addition, treatment with the PD-1 blocker did not induce IFNγ production on T98G. Moreover, IL-6 was abundantly present in supernatants of U87MG cells co-cultured with GiNKs (1:1, 5.6 ± 0.4 ng/mL) and GiNKs treated with the PD-1 blocker (5.2 ± 0.4 ng/mL). IL-6 was also abundantly present in the supernatants derived from T98G cells co-cultured with GiNKs (1:1, 7.7 ± 0.7 ng/mL) and GiNKs treated with the PD-1 blocker (8.3 ± 1.2 ng/mL). Moreover, the addition of the PD-1 blocker did not change the production of the IL-6. Elevated RANTES was detected in the supernatants from the cultures of effector cells alone (0.3 ng/mL). RANTES was also detected in the supernatants of cells with an E:T ratio of 1:1 compared to effector-only conditions. The addition of the PD-1 blocker did not enhance or attenuate the production of the RANTES (Figure 4).

### 2.6. Effects of GiNK Treatment in Combination with a PD-1 Blocker against a Subcutaneous Tumor Derived from GBM-like Cells

**Figure 5:**
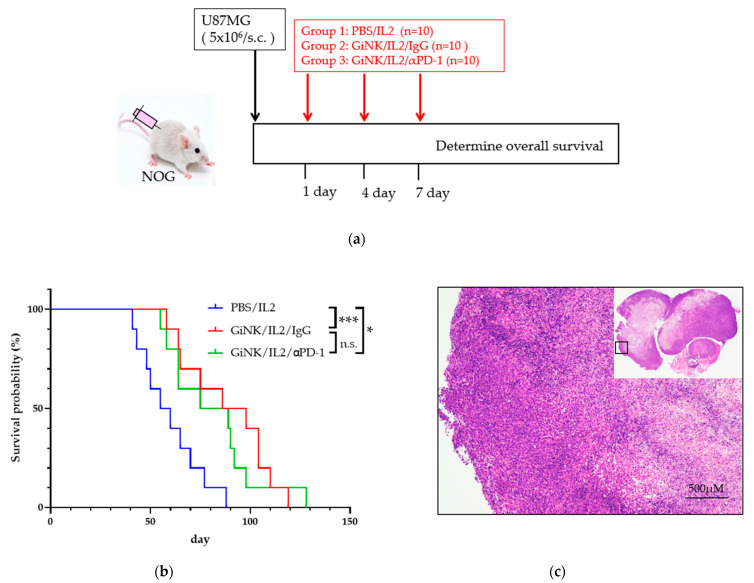
Effects of the GiNKs when used in combination with a PD-1 blocker against subcutaneous tumors derived from GBM-like cells. (**a**) Picture showing the experimental design. A total of 5 × 10^6^ U87MG cells were subcutaneously injected into the backs of NOG mice. The mice were then randomly assigned to three subcutaneous injection groups: PBS/IL-2 (10,000 IU/mL), GiNKs (10^7^)/IL-2 /IgG (40 µg), or GiNK/IL-2/PD-1 blocker (40 μg). The cells and drugs were injected intravenously via the retro-orbital sinus (retro-orbital injection) (**b**) The graph shows the Kaplan–Meier curve. Blue, red, and green lines represent the PBS/IL2 (*n* = 10), GiNK/IL-2/IgG (*n* = 10) and GiNK/IL2/PD-1 blocker (*n* = 10) groups, respectively. Statistical significance was determined using a Log-rank test. *** and * indicate *p* < 0.005 and *p* < 0.05, respectively. Not significant indicates n.s. (**c**) The picture shows hematoxylin & eosin stained a part of subcutaneous tumor derived from U87MG cells. (Upper right: entire subcutaneous tumor).

Next, we studied the anti-tumor effects of GiNKs with and without treatment with a PD-1 blocker on U87MG cells in NOG mice in vivo. To this end, NOG mice were subcutaneously inoculated with U87MG cells followed by three injections into the intravenous via retro-orbital sinus (days 1, 4, and 7) with PBS (control), GiNKs alone, or GiNKs with a PD-1 blocker (Figure 5a).

The mice treated with the GiNKs with and without the PD-1 blocker were associated with a significantly longer survival time compared to the PBS control group (*p* < 0.05). However, the presence of the PD-1 blocker did not influence the survival time (Figure 5b). The results of the histochemical analysis showed that the tumors from both groups exhibited a pseudopalisading pattern of tumor cells surrounding necrosis, which is a human GBM-like histological feature (Figure 5c).

## 3. Discussion

### 3.1. Current Status of Immunotherapy for GBM

Despite an aggressive standard of care regimen consisting of maximal surgical resection followed by combination radiation and chemotherapy, the prognosis of GBM patients remains poor, with a high recurrence rate and a median OS of less than 2 years [33]. Following the success of ICBs in melanoma and non-small cell lung cancer, interest in the use of immunotherapy as an alternative treatment approach for GBM has rapidly increased in recent years [31]. However, in GBM, immunotherapy, including vaccine therapy, viral therapy, and other cell-based therapies, has had a minimal impact on the OS [38,39]. Moreover, GBM has been proven to be highly resistant to standard treatments due to a combination of tumor heterogeneity, adaptive expansion of resistant cellular subclones, evasion of immune surveillance, and manipulation of various signaling pathways involved in tumor progression and the immune response [38].

### 3.2. Characteristics of GiNKs

NK cells exhibit potent cytotoxic activity against tumor cells via the induction of apoptosis [7] and can remove the abnormal cells as part of the innate immune system [8,9]. Moreover, NK cells play a role in immune surveillance and inhibit tumor occurrence, proliferation, and metastasis [22,23]. We previously reported the expansion of human peripheral blood NK cells using a novel culture system for clinical application as GiNKs. Our method of selectively expanding autologous human NK cells is associated with the highest purity and largest expansion scale using an easy, chemically defined and feeder-free method. Moreover, we expanded NK cells from CD3+ T cell-depleted PBMCs, which both enhanced the purity of NK cells and prevented contamination with regulatory T cells (Tregs). It has been reported that a reduction in the function and number of Tregs is beneficial as an immunotherapy against malignant tumors. In addition, GiNKs were reported to exhibit a strong anti-tumor effect against GBM cell lines through inducing apoptosis in vitro [35]. TMZ is widely used as the most effective anti-cancer drug for GBM in the current standard therapy when used concomitantly with radiotherapy. Moreover, GiNK induces apoptosis in TMZ-sensitive and TMZ-resistant human GBM cells and enhances the TMZ-induced antitumor effects in different mechanisms [35]. Based on our findings, immunotherapy using GiNK might represent a promising novel treatment option for patients with GBM.

### 3.3. Role of the PD-1/PD-L1 Pathway on GBM

The cytotoxic function of NK cells is determined by the balance between activating and inhibitory receptor signals [13,14]. Several activating receptors of NK cells (e.g., NKG2D and DNAM-1) recognize their ligands expressed on GBM [15,16,20], and the ligation of activating receptors triggers cytotoxicity in NK cells [17]. In addition, the ligands of NK inhibitory receptors, including PD-1, NKG2A, and KIR2DL, are also associated with NK cell cytotoxicity against tumor cells [18,19,40]. In the present study, we focused specifically on the PD-1/PD-L1 axis as an immune check point. The level of PD-L1 expression in GBM patients from the TCGA data set was associated with the WHO grading of glioma. In addition, Grade 4 glioblastoma was associated with significantly higher PD-L1 expression compared to Grades 2 and 3. According to the aggressive character of glioma, the PD-1/PD-L1 pathway might exhibit higher activation under conditions of immunosuppression. In this study, the level of PD-1 expression on GiNKs harvested from healthy volunteers was extremely low. Pesce et al. reported that NK cells in ovarian cancer patients had a significantly higher level of PD-1 expression compared to that of healthy volunteers [41]. The expression of PD-1 might also be higher in the NK cells of cancer patients that were activated under cancer immuno-surveillance [42,43].

The role of the PD-1/PD-L1 pathway in GBM remains controversial. Huang et al. reported that a blockade of the PD-1/PD-L1 pathway enhanced the anti-tumor effect of mouse NK cells [44]. However, in a recent Phase 3 randomized clinical trial for recurrent GBM treated with bevacizumab or nivolumab, nivolumab failed to demonstrate superiority. However, patients with a methylated MGMT promoter, glioblastoma, and no baseline corticosteroid use may potentially derive a benefit from treatment with immune checkpoint inhibition. [34].

### 3.4. Antitumor Effect of GiNKs against GBM In Vitro and In Vivo

To the best of our knowledge, less attention has been paid to the use of an NK cell-based treatment for GBM. We previously expanded human peripheral blood NK cells harvested from healthy volunteers using the novel culture system for clinical application as GiNKs and reported a strong anti-tumor effect in vitro [35]. The results of the present study showed that GiNKs significantly induced the apoptosis of U87MG and T98G cells in the apoptosis detection assays; however, the presence of a PD-1 blocker did not induce apoptosis in either cell line. We also determined the levels of cytokines produced by GiNK cells upon exposure to U87MG and T98G cells. GiNKs exposed to GBM cells produced significantly higher levels of IFNγ, IL-6, and RANTES. These results support the previous finding that GiNKs have significant anti-tumor effects for GBM cells through the induction of apoptosis and production of several cytokines.

RANTES accumulates in T cells and regulates inflammation in several diseases, playing an active role in recruiting a variety of leukocytes (e.g., T cells, macrophages, eosinophils, and basophils) into inflammatory sites. In collaboration with certain cytokines released by T cells (e.g., IL-2 and IFNγ), RANTES also induces the activation and proliferation of specific NK cells to generate chemokine-activated killer cells [45].

IL-6 was substantially increased when GiNKs were co-cultured with GBM cells. In the cytokine assay with GiNKs and GBM cells, it was not possible to determine which cells produced IL-6. The study by Goswami et al. reported that U87MG cells expressed IL-6 and IL-6 receptors, whereas U87MG cells have an autocrine growth loop [46]. Previously, IL-6/signal transducer and activator of transcription 3 (STAT3) signaling was reported to support GBM cell growth and migration [47]. Moreover, an IL-6/STAT3/hypoxia-inducible factor 1 subunit alpha autocrine loop has been observed in GBM. In addition, GBM cancer stem cells have been found to respond to perturbations caused by hypoxia, the inhibition of STAT3 phosphorylation, and IL-6 stimulation [48]. Thus, IL-6 is involved in the formation and progression of GBM. Based on our findings, U87MG cells may have released IL-6; however, a flow cytometry-based analysis of cytokine-producing cells is required to verify this finding. On the other hand, we observed that GiNKs elicit direct cytotoxicity and release cellular immunity-related cytokines, a finding that is contrary to the role of IL-6. Therefore, we aimed to verify the antitumor effect of GiNKs in vivo.

In our in vivo experimental GBM model, NOG mice were subcutaneously inoculated with U87MG cells and subsequently injected with GiNKs intravenously via the retro-orbital sinus three times (days 1, 4, and 7). Treatment with GiNKs with or without a PD-1 blocker exhibited a significantly longer survival time compared to that of the control group. However, there was no significant additional effects on the survival time in the group that received the GiNKs with the PD-1 blocker. Overall, the findings of our study suggest that the intravenous injection of GiNKs is highly effective against subcutaneously injected GBM cells independent of the PD-1/PD-1 pathway. There is a possibility that the GBM immunosuppression system against NK cells will add increased complexity through evasion via another pathway. 

There are several limitations associated with the present study. First, we used peripheral blood harvested from healthy volunteers. Typically, the expansion of NK cells from the blood of cancer patients is challenging due to the possibility of immune exhaustion following various types of chemotherapy. Second, we evaluated the anti-tumor effect of intravenously transferred GiNKs in a subcutaneous injection GBM model. It is possible that the adoptively transferred GiNKs might exhibit limited persistence. Moreover, GiNKs may not infiltrate the tumor through the brain-blood barrier, or the tumors could develop mechanisms to evade NK cell surveillance in the brain. Thus, our findings warrant validation in an intra-cranially injected model of GBM and perform a first in-human trial in future studies. Third, we have to pay attention to a natural immune system in mice before the injection of NK cells, even though we adopted the NOG mice in this experiment. We think that it is ideal to use CD34-humanized mice or PBMC-reconstituted mice, this setting would better inform on the efficacy of the combination since a PD-1 blocker would then target T cells present in the tumor. Moreover, we did not evaluate the effect of a PD-L1 blocker in vitro and in vivo. This might synergize better than PD1 alone to eradicate the tumors in combination with GiNKs.

## 4. Conclusions

We successfully demonstrated that ex vivo expanded and activated NK cells had an anti-tumor effect for GBM cells in vitro and vivo assays. Our NK cells prolong the overall survival of NOG mice subcutaneously injected with GBM-like cells. PD-1 blocking antibodies did not have an additive effect with our NK cells for prolonging survival in our xenograft model in GBM. Our findings reveal that our NK cells are less affected by the PD-1/PD-L1 immune evasion axis in GBM. In the future study, the anti-tumor effect of our NK cells in an intra-cranially injected model of GBM should be warranted.

## 5. Materials and Methods

### 5.1. Ethics

This study was approved by the ethics committee of Nara Medical University (approval number: 1058). All procedures in studies involving human participants were performed in accordance with the ethical standards of the institutional and/or national research committee and in line with the 1964 Declaration of Helsinki and its later amendments or comparable ethical standards. Informed consent was obtained from all healthy volunteers included in the study. We collected 8 mL of heparinized peripheral blood obtained from 3 healthy volunteers.

### 5.2. Reagents

Nivolumab as the PD-1 blockade agent was provided as a gift from Ono Pharmaceutical Co., Ltd., Osaka, Japan.

### 5.3. GBM Cell Lines

We obtained the human GBM cell line, T98G (CRL-1690), and human GBM-like cell line, U87MG (HTB-14), from the American Type Culture Collection (ATCC; Manassas, VA, USA). The cell lines were maintained in Dulbecco’s modified Eagle’s medium (DMEM; Life Technologies, Carlsbad, CA, USA) supplemented with 10% heat-inactivated fetal bovine serum (FBS; MP Biomedicals, Tokyo, Japan), 100 U/mL penicillin, and 100 µg/mL streptomycin (Life Technologies) at 37 °C in a humidified 5% CO_2_-containing atmosphere.

### 5.4. Induction of GiNKs

CD3-depleted peripheral mononuclear cells (PBMCs) were isolated using a RosetteSepTM Human CD3 Depletion Cocktail (STEMCELL Technologies, Vancouver, BC, Canada). CD3-depleted PBMCs were placed in a T25 culture flask (Corning, Steuben, NY, USA) containing AIM-V medium (Life Technologies, New York, NY, USA) supplemented with 5% autologous plasma, 50 ng/mL recombinant human (rh) interleukin (rhIL)-18 (Medical and Biological Laboratories Co., Ltd.; MBL, Nagoya, Japan), and 3000 IU/mL rhIL-2 (Novartis, Basel, Switzerland) at 37 °C in a humidified 5% CO_2_-containing atmosphere for 14 days. AIM-V supplemented with 3000 IU/mL IL-2 was replenished as necessary.

### 5.5. Analysis of Cell Surface PD-1 and PD-L1 Expression

Cells were stained with the appropriate antibodies and analyzed using a BD FACSCalibur flow cytometer (BD Biosciences, Franklin Lakes, NJ, USA) and CellQuest software ver 6.0 (BD Biosciences) and FlowJo ver 10 (BD Biosciences). The following antibodies were used: PE-conjugated anti-human CD279 (PD-1) (clone: EH12.2H7, Bio Legend, San Diego, CA, USA); PE-conjugated anti-human CD274 (PD-L1) (B7-H1, Bio Legend, San Diego, CA, USA); PE-conjugated mouse IgG2b (MPC-11, Bio Legend); AlexaFluor488-conjugated anti-human CD56 (B159, BD Pharmingen, Franklin Lakes, NJ, USA); PE-conjugated mouse IgG1 (MOPC-21, Bio Legend); and AlexaFluor488-conjugated mouse IgG1 (MOPC-21, BD Pharmingen). PD-1 expression on human-expanded γδT cells. The preparation of the γδT cells was described previously [49]. Briefly, human PBMCs were placed in a T25 culture flask containing AIM-V medium supplemented with 5% autologous plasma, 1 μM zoledronate (Novartis Pharma K.K., Tokyo, Japan) and 200 IU/mL rh IL-2. The cultures were maintained and expanded in AIM-V medium supplemented with 200 IU/mL rhIL-2 for 14 days. The expanded cellular population was stained with FITC-conjugated anti-TcR γδ (11F12, IgG BD Biosciences) and PE-conjugated PD-1 or PE-conjugated IgG.

### 5.6. Apoptosis Detection Assays

We performed apoptosis detection assays using an MEBCYTOTM Apoptosis Kit (MBL), in accordance with the manufacturer’s instructions. Briefly, GBM cell lines were exposed to GiNKs at E:T ratios of 0.5:1, 1:1, and 2:1 in the presence or absence of 10 μg Nivolumab for 10^7^ GiNKs for 4 h. Following the incubation, the floating cells were washed, and adherent cells were trypsinized with PBS, stained with Annexin V–FITC, and maintained at room temperature for 15 min in the dark. The stained cells were analyzed using a FACSCalibur flow cytometer and CellQuest Pro software ver 6.0. Notably, the NK cells were excluded by electronic gating based on the forward-scatter and side-scatter characteristics. The frequency of the Annexin V-positive populations was defined as apoptotic cells, as previously described [35,50].

### 5.7. Cytokine Detection

A Human Cytometric Bead Array Flex Set System (BD Biosciences, San Jose, CA, USA) was used to determine cytokine production in GiNKs cultured with GBM cells. GBM cells (U87MG or T98G cell lines) were co-cultured at ratios of 1:1 for 24 h with GiNKs. Four tests for cytokine detection were performed in each GBM cell. They included the group with GBM cells alone, with GiNKs alone, with GBM cells/GiNKs/IgG, and with GBM cells/GiNKs/PD-1 blocker. The concentrations of interferon-γ (IFNγ: 558269), interleukin-6 (IL-6: 558276), and regulated on activation, normal T-cells expressed and secreted (RANTES: 558324) in the supernatant were determined. The experiment was performed three times. The assays were performed according to the manufacturer’s instructions. Data were acquired on a BD FACSMelody flow cytometer (BD Biosciences).

### 5.8. In Vivo Xenograft Assay

The in vivo xenograft assay was performed as described previously [50,51]. Briefly, nonobese diabetes (NOD)/severe combined immunodeficiency (SCID)/γcnull (NOG) mice were purchased from the Central Institute for Experimental Animals (Kanagawa, Japan). All animal experiments were approved by the Institutional Animal Care and Use Committee of Nara Medical University. A total of 5 × 10^6^ U87MG cells were subcutaneously injected into the backs of 8-week-old female NOG mice. The mice were randomly assigned to three subcutaneous (s.c.) injection groups: PBS/IL-2 (10,000 IU/mL), GiNK (10^7^)/IL-2 (10,000 IU/mL)/IgG (40 µg), GiNK (10^7^)/IL-2 (10,000 IU/mL) PD-1 blocker (40 µg), respectively. The cells and drugs were suspended in 250 µL PBS and injected intravenously via the retro-orbital sinus. Retro-orbital injections were performed using a 29 G needle under isoflurane anesthesia as previously reported [52].

### 5.9. Histochemical Analysis

The subcutaneous tumors were fixed with 10% neutral-buffered formalin and embedded in paraffin. After sectioning, 5-µm-thick sections were placed on glass slides and stained with hematoxylin and eosin (HE). Photographs were taken using an BX-710 (KEYENCE, Osaka, Japan) at 40× magnification.

### 5.10. Statistical Methods

Data are presented as the mean ± standard error. Statistical analyses were performed using Prism 8 (GraphPad Software Inc., San Diego, CA, USA). Statistically significant differences were determined using a Turkey’s Honest Significant difference test and one-way analysis of variance (ANOVA) followed by Tukey’s test and two-way ANOVA followed by Sidak test. *p* < 0.05 was considered statistically significant. Kaplan–Meier curves was also produced using Prism 8. Statistically significant differences were determined using a log-rank test.

## Data Availability

The data supporting the findings of this study are available from the corresponding author upon reasonable request.

## Supplementary Materials

The following are available online at https://www.mdpi.com/article/10.3390/ijms22189975/s1.
