# Supplementary figures and images for: Ex Vivo Expanded and Activated Natural Killer Cells Prolong the Overall Survival of Mice with Glioblastoma-like Cell-Derived Tumors
Source: Int J Mol Sci. 2021 Sep 15;22(18):9975. doi: 10.3390/ijms22189975 (PMC8472834; doi:10.3390/ijms22189975)

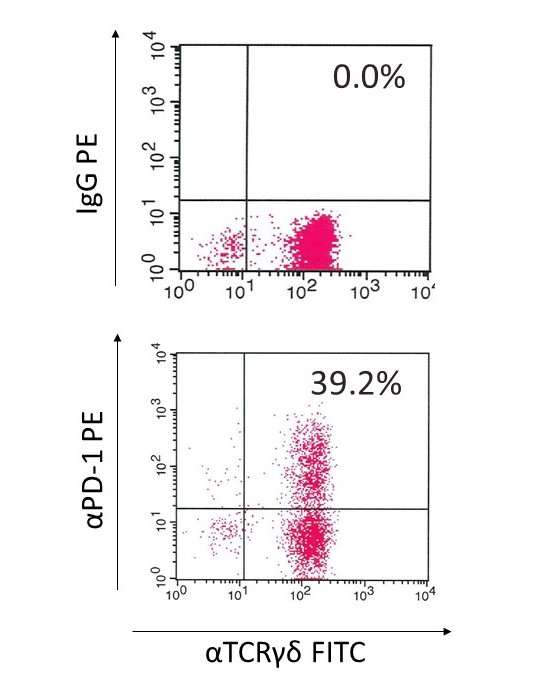

Supplement: Supplementary file 1 [file ijms-22-09975-s001.zip › ijms-1330671-supplementary.jpg]
